# Supplementary material for: Multilocus sequence typing of Candida albicans oral isolates reveals high genetic relatedness of mother-child dyads in early life
Source: PLoS One. 2024 Jan 17;19(1):e0290938. doi: 10.1371/journal.pone.0290938 (PMC10793898; doi:10.1371/journal.pone.0290938)
Supplement: S1 Appendix — (DOCX) [file pone.0290938.s002.docx]

**Appendix 1 – Additional Description for Methods Section**

**Oral examination and data/sample collection**

Dental caries of the mothers and children was assessed using the decayed, missing, and filled teeth (DMFT/dmft) method according to the codes recommended by the World Health Organization's Oral Health Surveys - Basic Methods, 4th edition in 1997 (1). Dental plaque was evaluated using the plaque index described by Löe in 1967 (2), while oral mucosa was assessed using clinical diagnostic criteria for oral candidiasis and defined as pseudomembranous/erythematous forms (3). Sample collection was conducted at Highland Family Medicine or Perinatal Dental Clinic at Eastman Institute of Oral Health, University of Rochester. Sample collection took place throughout the day and participants were instructed not to eat or drink beverages 30 minutes before oral sample collection. Saliva samples of approximately 2 ml were collected from the mothers by spitting into a sterilized 50ml centrifuge tube, while SalivaBio Infant's Swab was used to collect saliva samples from the infants at each visit. Supragingival plaque from the whole dentition was collected using a sterilized periodontal scaler from both mothers and children (12-, 18-, and 24-month visits) (4), and the plaque samples were suspended in 1ml of a 0.9% sodium chloride solution in a sterilized Eppendorf tube. After collection, the clinical samples (saliva/plaque) were stored on ice and

transferred to the lab located at Center of Oral Biology, University of Rochester within 2 hours for laboratory testing.

**References**

1. Organization. WH. Oral health surveys - basic methods, 4th ed.: Geneva: World Health Organization; 1997.

2. Loe H. The Gingival Index, the Plaque Index and the Retention Index Systems. J Periodontol. 1967;38(6):Suppl:610-6.

3. Coronado-Castellote L, Jimenez-Soriano Y. Clinical and microbiological diagnosis of oral candidiasis. J Clin Exp Dent. 2013;5(5):e279-86.

4. Xiao J, Moon Y, Li L, Rustchenko E, Wakabayashi H, Zhao X, et al. Candida albicans Carriage in Children with Severe Early Childhood Caries (S-ECC) and Maternal Relatedness. PLoS One. 2016;11(10):e0164242.
