# Supplementary material for: Multilocus sequence typing of Candida albicans oral isolates reveals high genetic relatedness of mother-child dyads in early life
Source: PLoS One. 2024 Jan 17;19(1):e0290938. doi: 10.1371/journal.pone.0290938 (PMC10793898; doi:10.1371/journal.pone.0290938)
Supplement: S1 Table — (DOCX) [file pone.0290938.s003.docx]

**S1 Table. Number and source of *C. albicans* isolates used in the MLST analysis.**

| Visit number | 1 (3^rd^ trimester mother) | 2 (1 month infant) | 3 (2 months infant) | 4 (4 months infant) | 5 (6 months infant) | 6 (12 months infant) | 7 (18 months infant) | 8 (24 months infant) | Total |
| --- | --- | --- | --- | --- | --- | --- | --- | --- | --- |
| Saliva isolates | 49 | 10 | 25 | 19 | 24 | 35 | 32 | 24 | 218 |
| Plaque isolates | 2 | N/A | N/A | N/A | 0 | 1 | 2 | 4 | 9 |
| Total | 51 | 10 | 25 | 19 | 24 | 36 | 34 | 28 | 227 |

N/A: Not applicable
