# Supplementary material for: Multilocus sequence typing of Candida albicans oral isolates reveals high genetic relatedness of mother-child dyads in early life
Source: PLoS One. 2024 Jan 17;19(1):e0290938. doi: 10.1371/journal.pone.0290938 (PMC10793898; doi:10.1371/journal.pone.0290938)
Supplement: S2 Table — (DOCX) [file pone.0290938.s004.docx]

**Supplemental Tables**

**S2 Table. Characteristics of the seven housekeeping loci used in *C. albicans* MLST.**

|  | **Locus** | **Bases sequenced** | **Genotypes*** | **SNPs*** |
| --- | --- | --- | --- | --- |
|  | *AAT1a* | 373 | 204 | 72 |
|  | *ACC1* | 407 | 126 | 51 |
|  | *ADP1* | 443 | 184 | 68 |
|  | *MPIb* | 375 | 185 | 65 |
|  | *SYA1* | 391 | 253 | 158 |
|  | *VPS13* | 403 | 345 | 60 |
|  | *ZWF1a* | 491 | 324 | 78 |
| Total | 7 | 2883 | 1621 | 552 |

***** Data presented as of March 2, 2023
