# Supplementary material for: Multilocus sequence typing of Candida albicans oral isolates reveals high genetic relatedness of mother-child dyads in early life
Source: PLoS One. 2024 Jan 17;19(1):e0290938. doi: 10.1371/journal.pone.0290938 (PMC10793898; doi:10.1371/journal.pone.0290938)
Supplement: S3 Table — (DOCX) [file pone.0290938.s005.docx]

**Supplemental Tables**

**S3 Table. Novel allele sequences for each locus identified in our study.**

| **Locus** | **Number of novel alleles identified** | **Assigned allele number** |
| --- | --- | --- |
| ***AAT1a*** | 9 | 196, 197, 198, 199, 200, 201, 202, 203, 204 |
| ***ACC1*** | 3 | 124, 125, 126 |
| ***ADP1*** | 5 | 180, 181, 182, 183, 184 |
| ***MPIb*** | 2 | 184, 185 |
| ***SYA1*** | 6 | 247, 248, 249, 250, 251, 252 |
| ***VPS13*** | 13 | 319, 320, 322, 324, 326, 327, 328, 330, 332, 333, 334, 335, 336 |
| ***ZWF1b*** | 13 | 302, 304, 305, 306, 307, 308, 311, 312, 315, 316, 317, 318, 320 |

Our study identified several novel allele sequences for each locus examined, expanding our understanding of the genetic diversity within the studied population. A total of 51 new allele sequences were identified and submitted to the PubMLST database for curation by Dr. Marie-Elisabeth Bougnoux. Each sequence was assigned a unique number for identification.
