# Supplementary material for: Multilocus sequence typing of Candida albicans oral isolates reveals high genetic relatedness of mother-child dyads in early life
Source: PLoS One. 2024 Jan 17;19(1):e0290938. doi: 10.1371/journal.pone.0290938 (PMC10793898; doi:10.1371/journal.pone.0290938)
Supplement: S1 Fig — The evolutionary pathway of C. albicans is indicated in italicized typeface on a lighter grey background. Taxonomic classifications are indicated in plain typeface on a darker grey background. The summary was adapted from in addition to using the online databases http://www.catalogueoflife.org/ and http://www.mycobank.org/. (DOCX) [file pone.0290938.s006.docx]

**Supplemental Figures**
**S1 Fig. Summary of the current understanding of the ancestry and phylogeny of *Candida* *albicans*.** The evolutionary pathway of *C. albicans* is indicated in italicized typeface on a lighter grey background. Taxonomic classifications are indicated in plain typeface on a darker grey background. The summary was adapted from McManus et al., 2014 (8) in addition to using the online databases http://www.catalogueoflife.org/ and http://www.mycobank.org/.
