# Supplementary material for: Multilocus sequence typing of Candida albicans oral isolates reveals high genetic relatedness of mother-child dyads in early life
Source: PLoS One. 2024 Jan 17;19(1):e0290938. doi: 10.1371/journal.pone.0290938 (PMC10793898; doi:10.1371/journal.pone.0290938)
Supplement: S2 Fig — The proportion of children who were exclusively breastfed decreased steadily from 36% at one month to 4% at two years. Conversely, the proportion of children who were exclusively bottle-fed nearly doubled from 29% at one month to 56% at six months, then remained stable from 6 to 12 months before sharply declining at 18 months. Night breastfeeding gradually decreased from 68% at one month to 3% at the age of two years. During the first six months, approximately 70% of children were fed with a bottle at night, but this declined sharply after six months and reached 3% by the time they were 18 months old. (DOCX) [file pone.0290938.s007.docx]

**Supplemental Figures**

|  |  |
| --- | --- |
|  |  |
|  |  |
|  |  |
|  |  |
|  |  |
|  |  |

**S2 Fig. Child feeding pattern during the first two years of life.**

The proportion of children who were exclusively breastfed decreased steadily from 36% at one month to 4% at two years. Conversely, the proportion of children who were exclusively bottle-fed nearly doubled from 29% at one month to 56% at six months, then remained stable from 6 to 12 months before sharply declining at 18 months. Night breastfeeding gradually decreased from 68% at one month to 3% at the age of two years. During the first six months, approximately 70% of children were fed with a bottle at night, but this declined sharply after six months and reached 3% by the time they were 18 months old.
